# Polylactic Acid/Cerium Fluoride Films: Effects of Cerium Fluoride on Mechanical Properties, Crystallinity, Thermal Behavior, and Transparency

**DOI:** 10.3390/ma14174882

**Published:** 2021-08-27

**Authors:** Yincai Wu, Xintu Lin, Jinlei Li, Chuanxiang Zhang, Yuejun Liu, Lijun Song, Xihai Hao, Fenglong Lin, Shenglong Wang, Tungalag Dong

**Affiliations:** 1 Key Laboratory of Advanced Packaging Materials and Technology of Hunan Province, School of Packaging and Materials Engineering, Hunan University of Technology, Zhuzhou 412007, China; xmwuyincai@fjirsm.ac.cn (Y.W.); xinxlin@126.com (X.L.); cxzhang7335@126.com (C.Z.); linlong9386@163.com (F.L.); 2 Xiamen Key Laboratory of Rare Earth Photoelectric Functional Materials, Xiamen Institute of Rare Earth Materials, Chinese Academy of Sciences, Xiamen 361021, China; 13997894639@163.com (J.L.); xmwangshenglong@fjirsm.ac.cn (S.W.); 3 Key Laboratory of Design and Assembly of Functional Nanostructures, Fujian Provincial Key Laboratory of Nanomaterials, Fujian Institute of Research on the Structure of Matter, Chinese Academy of Sciences, Fuzhou 350002, China; 4 School of College of Food Science and Engineering, Inner Mongolia Agricultural University, Hohhot 010000, China; dongtlg@imau.edu.cn

**Keywords:** PLA, CeF_3_, crystallinity, visible light transmittance, transparency

## Abstract

PLA is widely used in the field of disposable products for its good transparency, high strength, high modulus, and good processing performance. However, the crystallization rate and crystallinity of PLA are weak. In actual production, the PLA products that are typically obtained are amorphous with poor heat resistance, which greatly limits the application range of PLA products. Finding an effective nucleating agent to improve the transparency of PLA has been a hot topic in research. This study found that Cerium fluoride (CeF_3_) can effectively improve the crystallinity of PLA/CeF_3_ (P/F) films. When the content of CeF_3_ in PLA was 1 wt %, the retention ratio of visible light transmittance was 82.36%, the crystallinity was 29.8%, and the tensile strength was 59.92 MPa. Compared to pure PLA, the crystallinity of P/F1 increased by 56% and tensile strength increased by 8.76%. This study provided an alternative scheme that maintained the PLA film’s transparency and improved the crystallinity of PLA, which significantly expanded the application of PLA.

## 1. Introduction

Polylactic acid (PLA) is a kind of aliphatic thermoplastic polyester and biodegradable biobased polymer [1,2,3,4,5,6,7,8,9,10]. In contrast to traditional fossil-based polymers, it is one of the most promising biobased polymers that plays an important role in the polymer market [11,12,13,14,15,16]. It can effectively prevent and alleviate environmental problems such as “white pollution” [1,2,3,4,17]. Moreover, PLA is one of the most suitable candidate materials to replace polystyrene (PS), polyethylene terephthalate (PET), polyethylene (PE) and polypropylene (PP) [18,19,20,21,22,23]. Due to its good transparency, high strength, high modulus, and good processing performance, PLA has broad applications in the field of disposable products, such as food packaging, tableware, water cup, water bottle, etc.

However, the practical applications of PLA are limited because of its brittleness, poor toughness, low crystallinity, and fast crystallization speed [24,25]. Some physical properties, such as the mechanical, heat resistance and barrier properties of PLA, are correlated to its crystallinity [26]. So far, many studies have been conducted on the crystallization behavior of PLA [27,28,29,30]. Generally, the crystallinity will be improved by chemical and physical methods. Chemical methods usually refer to manipulation of the PLA polymer structure at the molecular level, while physical methods are performed by expanding the nucleating agent to the crystalline region in the polymer matrix [31,32]. At present, there are two kinds of nucleating agents: organic nucleating agents and inorganic nucleating agents. Organic nucleators mainly include hydrazide, aliphatic amide, amide, etc., such as ethylenebisearamide [33], 1,2-hydroxystearamide [34], tmc-328 [35,36,37] and *N*,*N*′-bis(benzoyl) Diacylhydrazine hexanedioate [38], algal residue nanocellulose, etc. [39]. Inorganic nucleators mainly include montmorillonite, talc powder, mica, calcium carbonate, carbon nanotubes, graphene, magnesium oxide, etc. [40,41,42,43,44,45]. In some previous reports, adding a nucleating agent to PLA can not only increase crystallinity, but also improve its mechanical properties and thermal stability [44,45].

Due to their large ionic radius and unique electronic structure, rare earth elements exhibit high coordination number and strong coordination ability, especially with strong acid elements such as oxygen and sulfur [46]. In this paper, CeF_3_ as the second phase was added to polylactic acid by means of coordinated interactions; cerium ions will play a special role in tuning the crystallinity and modifying the physical properties of PLA.

## 2. Materials and Methods

### 2.1. Materials

Poly(lactic acid) (PLA) (PLA, 3001D; MFR, 22 g/10 min; specific gravity, 1.24; melting point, 173 °C; crystallinity, 19%; optical purity, ≥96%; clarity, transparent) was obtained from Nature Works in pellet form. CeF_3_ (boiling point, 2300 °C; melting point, 1640 °C; molecular weight, 197.1112; purity, 99.9%; specific density, 0.162; granularity, D50 = 1.16 μm) was obtained from the Hunan Institute of Rare Earth Materials.

### 2.2. Preparation of PLA/CeF_3_ (P/F) Blends and Films

**Figure 1:**
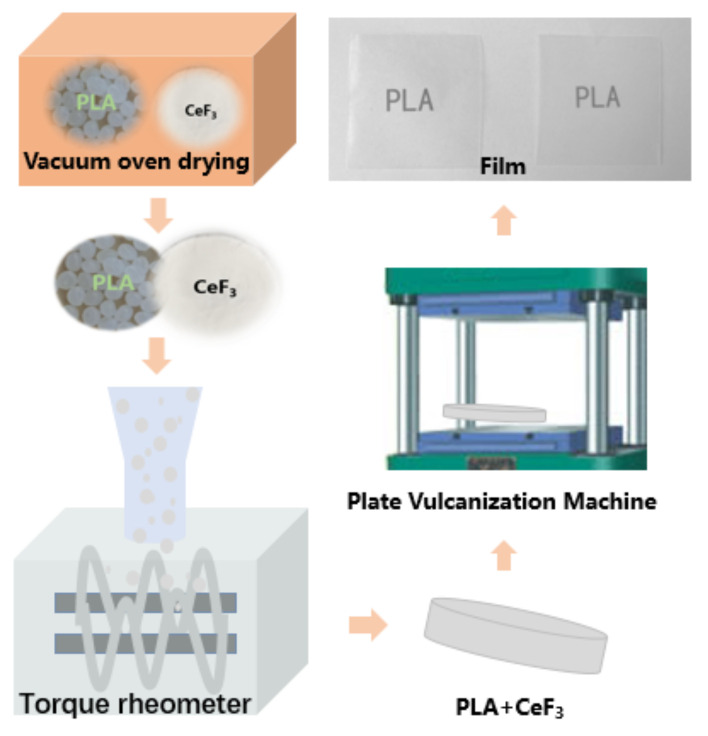
Schematic illustration of the preparation of P/F films.

PLA and CeF_3_ were dried at 70 °C for 4 h in a vacuum oven before further processing. PLA/CeF_3_ (P/F) blends were prepared by using a torque rheometer (RT01-06/02, Guangzhou, China) in mass ratios of 100/0, 99.5/0.5, 99/1, 98/2, 97/3, and 96/4; the formulation of P/F mixtures is shown in Table 1. The rotating speed was 60 rpm at 180 °C for 5 min. Then, all the samples were hot-pressed onto 0.1 mm-thick sheets at 180 °C with a pressure of 15 MPa of 2 min, and then, cooled down to room temperature; samples were cold-pressed again, and the final P/F films were obtained. A schematic illustration of the preparation of P/F films is shown in Figure 1.

### 2.3. Characterizations

The tensile properties of the P/F films were tested by a universal material testing machine (Instron 2365, Darmstadt, Germany) at a crosshead speed of 50 mm/min according to the ASTM D638-2008 standard. The P/F films were conditioned at room temperature in a 50% relative humidity-controlled environment for at least 24 h prior to being tested. At least five runs for each sample were measured, and the results were averaged. Then, the tensile strength, Young’s modulus and elongation at break of the samples were obtained. A Fourier transform infrared spectrophotometer (Nicolet iS 50, Madison, WI, USA) was used to investigate the possible intermolecular interaction between PLA and CeF_3_. The average value was obtained from 32 scans in the standard wave-number range of 500 to 4000 cm^−1^. A UV-visible-near infrared light spectrophotometer (Cary 5000, Santa Clara, CA, USA) was used to characterize the transmittance of the P/F films in the visible light region. The test wavelength range is 200–800 nm. Field emission scanning electron microscopy (FESEM) (Apreo S, Waltham, MA, USA) was used to characterize the cross-section and surface phase morphology of the P/F films. A layer of platinum was sputter-coated uniformly over all the fractured surfaces before FESEM observation. X-ray diffraction (Miniflex 600, Akishima-shi, Japan) was used to characterize the crystallinity of the P/F films. Measurements were performed over the ranges 5–70 °C, with a step of 0.05 °C and speed 10 °C/min. The thermal and crystallization behaviors of the PLA/CeF_3_ blends were studied by differential scanning calorimetry (TGA/DSC 1, Zurich, Switzerland) under nitrogen atmosphere. The weight of the samples varied from 5.0 to 10.0 mg. The samples were heated from 30 °C up to 190 °C at 10 °C/min (the first heat scan) and held at 190 °C for 3 min to eliminate their previous thermal history. Following this, the samples were chilled to 30 °C at the same rate, and then, heated again from 30 °C up to 190 °C at 10 °C/min (the second heating scan). The thermal stability of the P/F films was studied by thermogravimetric analyses. Samples of about 5 mg were placed in alumina crucibles and were measured in dynamic conditions, in the temperature range from 30 to 600 °C, with a heating rate of 10 °C/min, and a 50 mL/min Ar_2_ flow. Dynamic mechanical analysis was performed in a DMA instrument (DMA 1, Mettler Toledo, Zurich, Switzerland) in the tensile mode, based on the ASTM standard D4092. All samples were cut from the tensile bar specimens (40 mm × 10 mm × 0.1 mm). The temperature ranged from −20 to 100 °C, with a heating rate of 3 °C/min at an oscillating frequency of 1 Hz.

## 3. Results and Discussion

### 3.1. Mechanical Properties

**Figure 2:**
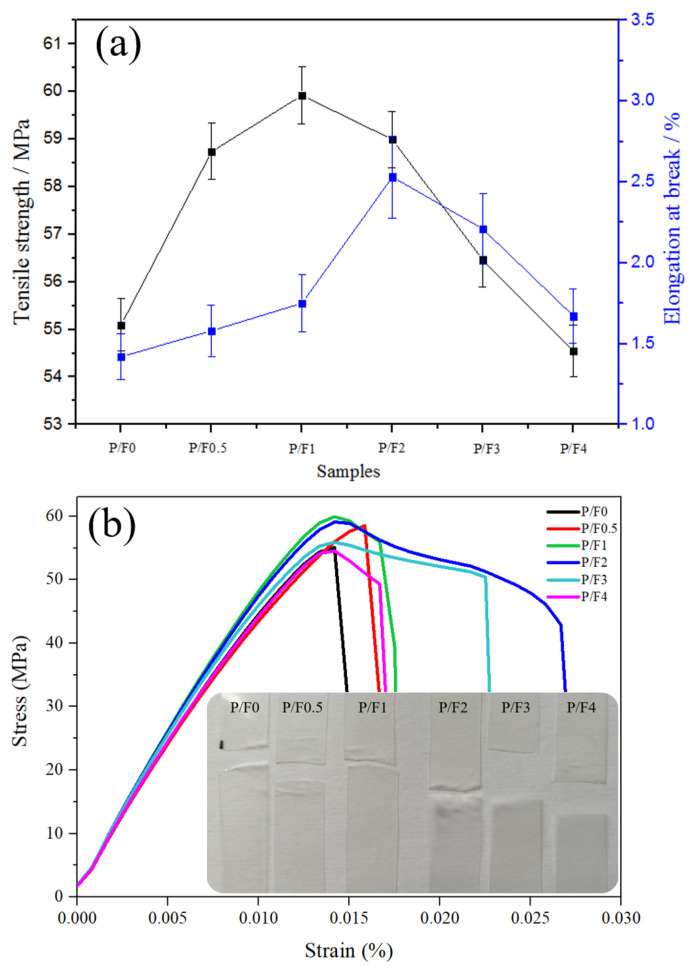
(**a**) Mechanical properties and (**b**) tensile stress–strain curves of P/F films.

As shown in Figure 2a, with the increase in CeF_3_ content, the tensile strength and elongation at break of the P/F films increased at first and then decreased. When the content of CeF_3_ was 1 wt %, the tensile strength of the P/F1 film was the highest, reaching from 55.09 to 59.92 MPa, which was 8.76% higher than that of pure PLA. When the content of CeF_3_ was 2 wt %, the elongation at break of the P/F2 film was the highest, reaching 2.53%, which was 78% higher than that of pure PLA. The tensile stress–strain curves of P/F films are shown in Figure 2b. When the content of CeF_3_ was 2 wt %, the overall effect of strengthening and toughening was better. This showed that CeF_3_ improved the ductibility ability of P/F film in the range of 0.5% to 2%.

### 3.2. Fourier Transform Infrared Spectrophotometer (FTIR)

The characteristic absorption peak of PLA included: −CH_3_ asymmetric stretching vibration peaks observed at 3000 and 1460 cm^−1^; symmetrical stretching vibration peaks were observed at 1380 cm^−1^. The stretching vibration peak of −C=O was observed at 1750 cm^−1^. −CH stretching and bending vibration peaks were observed at 2950 and 1360 cm^−1^, respectively. The stretching vibration peaks of −C−O−C− were 1260, 1180 and 1080 cm^−1^ [47,48,49].

**Figure 3:**
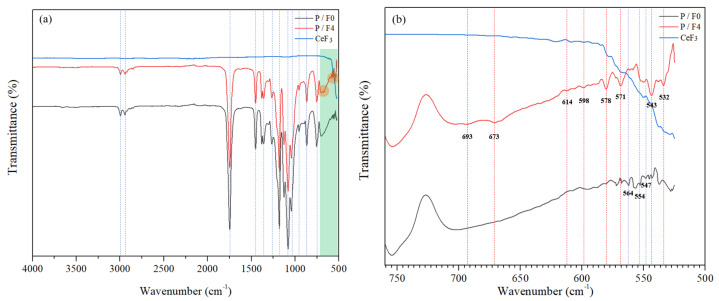
Infrared spectra of CeF_3_, P/F0 and P/F4 films. (**a**) Wavenumber range 500–4000 cm^−1^; (**b**) Wavenumber range 500–730 cm^−1^.

As shown in Figure 3, compared with pure PLA (P/F0), the spectral peak of P/F4 had several new characteristic peaks in the range of 500~700 cm^−1^. For example, these characteristic peaks (532, 543, 571, 578 and 598 cm^−1^) of P/F4 represent the coordination interactions that exist between cerium and oxygen atoms in PLA. In addition, new characteristic peaks also appeared in 673 and 693 cm^−1^. These two peaks were assigned to the vibration modes from the Ce-O band.

In general, the main characteristic peaks of PLA were retained, which proved that CeF_3_ does not change the main structure of PLA, but had a certain coordination effect and coupling relationship. The coordination effect and coupling relationship were conducive to enhancing the mechanical properties of PLA, which was also verified by the mechanical property test results in Figure 2.

### 3.3. UV–Vis Transmittance

In order to study the effect of CeF_3_ on the visible light transmittance of P/F films, the UV–vis spectral transmittance of P/F films was measured. The calculation formula of visible light transmittance is the following relationship (Equation (1)):

(1) $$T_{v}\;\%=\frac{T_{w760\;}\%+T_{w390}\;\%}{2}$$

where $T_{v}\;\%$ is the average visible light transmittance, $T_{w760}\;\%$ is the visible light transmittance at 760 nm, and $T_{w390}\;\%$ is the visible light transmittance at 390 nm.

**Figure 4:**
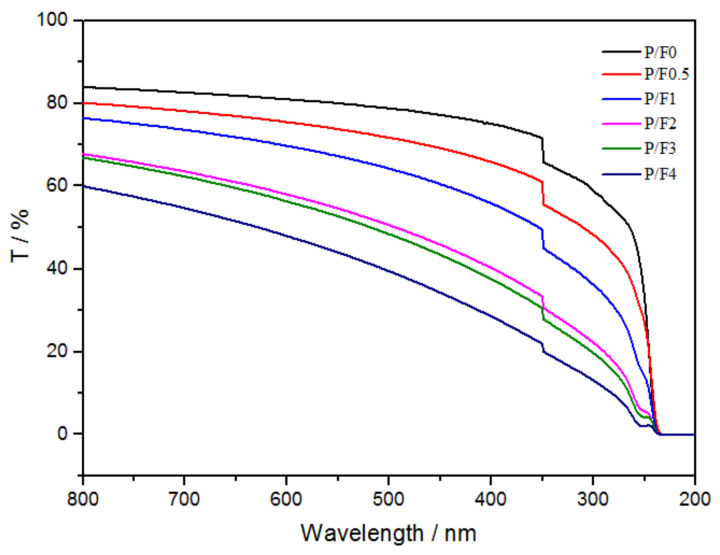
UV-vis transmittance of P/F films.

Figure 4 shows that the visible light transmittance of P/F films decreased slightly with the increase in the addition of CeF_3_ and the specific data, as shown in Table 2.

**Figure 5:**
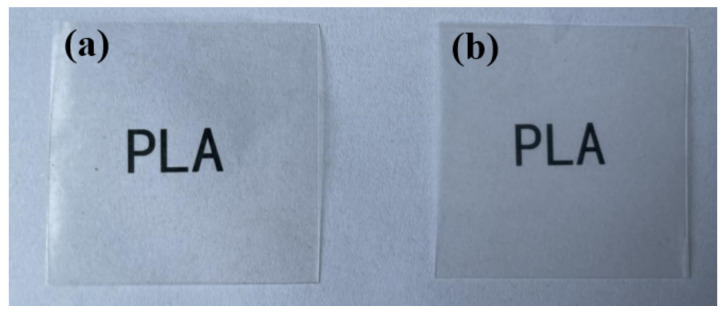
Pure PLA film (**a**) and P/F1 film (**b**) samples.

When the content of CeF_3_ in PLA was 1 wt %, the visible light transmittance retention ratio of the P/F1 film was 82.36%. Pure PLA film and P/F1 film samples are shown in Figure 5.

### 3.4. Morphological Properties

**Figure 6:**
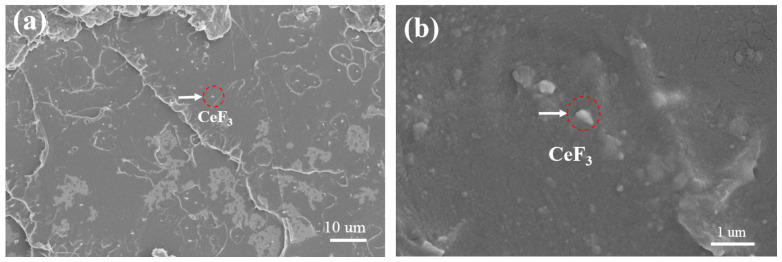
(**a**) Cross-section and (**b**) surface morphology of P/F2 films.

Figure 6 shows the cross-section and surface morphology of P/F films. The FESEM images show that CeF_3_ were well dispersed in the P/F films, and there were few cavities in the brittle cross-section, which indicated that the interface between CeF_3_ and PLA was good. This may be due to the formation of various coordination modes between cerium and oxygen in the hydroxyl and carboxyl groups of PLA, and the formation of layered, network polymers or infinite chain structure, which improved the compatibility of different components. It also indicated that the addition of CeF_3_ could tune the strength and toughness of PLA, which was consistent with the test results of mechanical properties.

### 3.5. X-ray Diffraction (XRD)

**Figure 7:**
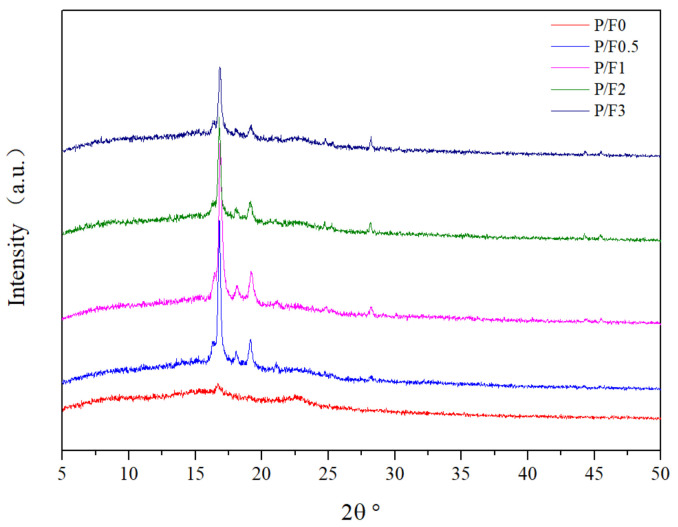
XRD patterns of P/F films annealed for 2 h at 110 °C.

In order to analyze the effect of CeF_3_ on the crystal structure of PLA, the annealed P/F films were analyzed by XRD. Figure 7 shows the XRD patterns of P/F films annealed for 2 h at 110 °C. There was a diffuse hump peak of P/F0 without annealing between 5 and 25 °C, indicating that the P/F0 was in the amorphous state. When P/F0 was annealed for 2 h, only a weak crystallization peak appeared. However, when PLA was added to CeF_3_, an obvious crystallization peak appeared. With the increase in CeF_3_ content, the crystallization peak intensity increased first and then decreased slightly, which proved that a certain amount of CeF_3_ was helpful to promote the crystallization of PLA.

### 3.6. Differential Scanning Calorimetry (DSC)

**Figure 8:**
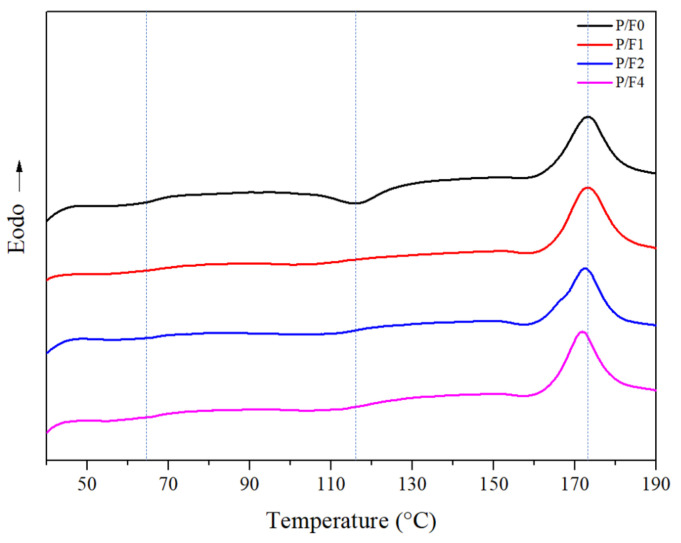
DSC second heating curves of P/F films.

DSC analysis was carried out to investigate the melting and crystallization behaviors of the P/F films. The DSC second heating curves of P/F films are shown in Figure 8, which displayed three main transitions successively: a glass transition, a cold crystallization exotherm, and a melting endotherm. The measured values of the phase transition parameters are summarized in Table 3. With the increase in CeF_3_ content, the glass transition temperature ($T_{g}$) of PLA hardly changed, suggesting that CeF_3_ did not change the glass transition temperature of PLA. However, the addition of CeF_3_ significantly decreased the cold crystallization temperature ($T_{cc}$) of PLA. When the CeF_3_ content increased from 0.5 wt % to 4 wt %, the $T_{cc}$ of P/F films decreased from 116.25 to 103.99 °C gradually and the $∆H_{c}$ gradually decreased from 10.97 to 1.87 J/g, indicating the addition of CeF_3_ could accelerate the crystallization of PLA. The degree of crystallization of the sample was evaluated from the heat evolved during crystallization by the following relationship (Equation (2)):

(2) $$X_{c}\;\%=\frac{∆H_{m}-∆H_{c}}{W_{PLA}\times ∆H_{m}^{0}}\times 100\;\%$$

where $X_{c}\;\%$ is the degree of crystallinity of the samples, $∆H_{m}$ is the heat of fusion of the PLA in the blend, $∆H_{c}$ is the enthalpy of cold crystallization of the PLA in the blend, $∆H_{m}^{0}$∆ is the heat of fusion for 100% crystalline PLA (93.1 J/g) [34], and $W_{PLA}$ is the weight fraction of PLA in the blend.

**Figure 9:**
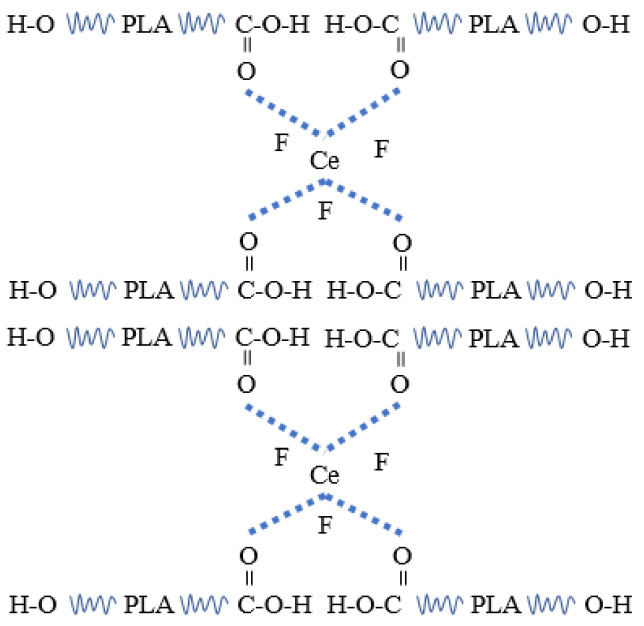
Schematic diagram of the interaction between CeF_3_ and PLA.

When the content of CeF_3_ in PLA was 1 wt %, the crystallinity was 29.8%, which was 56% higher than that of pure PLA. Other P/F films’ crystallinity is shown in Table 3. The crystallization ability increased likely because of the coordination interaction between PLA and CeF_3_ or heterogeneous nucleation in PLA, which might result in the ordered segmental arrangement of PLA chains. A schematic diagram of the interaction between CeF_3_ and PLA is shown in Figure 9.

### 3.7. Thermogravimetric Analyses (TGA)

**Figure 10:**
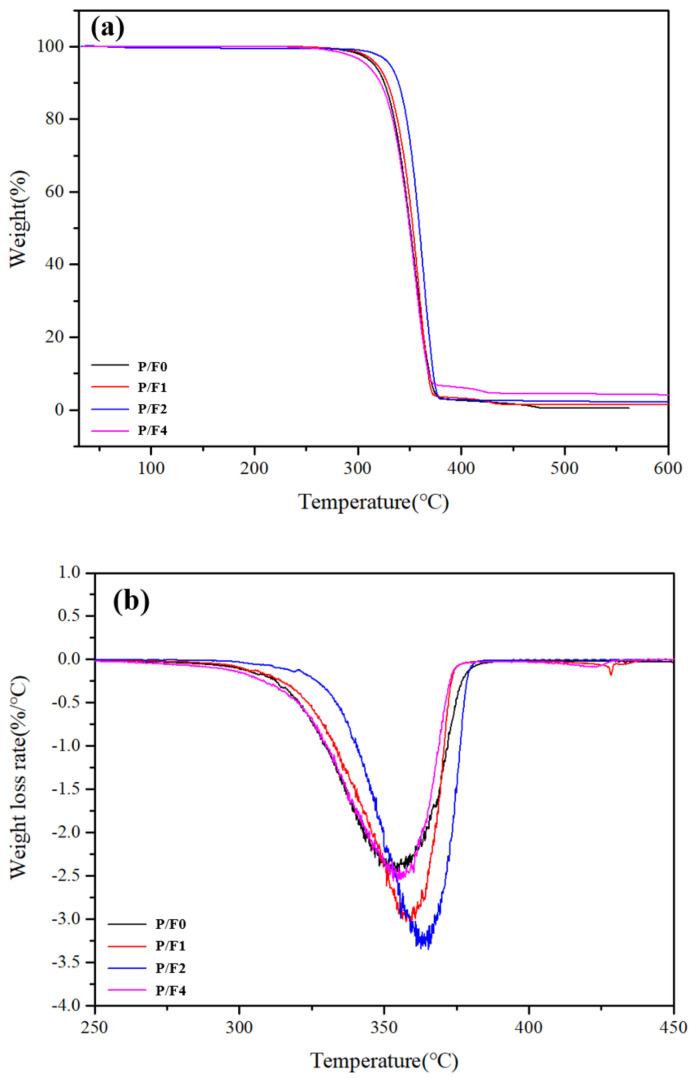
TGA (**a**) and DTG (**b**) of P/F films.

The thermal stability of PLA is critical, as this property is considered as the limiting factor for processing as well as for end-use applications [50]. Figure 10 shows the TGA thermograms of the P/F films, and Table 4 presents a summary of thermal performance, i.e., the initial, 5% mass loss, maximum mass loss and final residue at 500 °C. The mass was barely lost before 100 °C, proving that the moisture in PLA was removed by drying before further processing. The TGA curve was a flat period before 250 °C, then dropped at 280–380 °C suddenly, and then, tended to be stable. It was a typical one-step degradation reaction [51]. After 500 °C, P/F almost decomposed completely.

The initial thermal stability of the P/F film was like that of pure PLA. The TGA data of the P/F films are shown in Table 4. When the amount of CeF_3_ was 2 wt %, the initial degradation temperature of P/F2 films was 330.8 °C, and the maximum degradation temperature was 364.2 °C, which was 15.1 and 10.1 °C higher than that of pure PLA, respectively. When the amount of CeF_3_ was more than 2%, the initial decomposition temperature of the P/F1 film was lower than that of pure PLA, but the maximum degradation temperature was still higher than that of pure PLA. This was likely due to the effect of CeF_3_ on the interaction between PLA chains. When at a lower temperature, the movement ability of PLA molecular chains was limited and the effect of CeF_3_ on the interaction between PLA molecular chains was dominant. With the temperature increase, the movement ability of PLA molecular chains intensified, and the heterogeneous CeF_3_ particles promoted the crystallization of PLA. Consequently, the DSC test results were confirmed, resulting in the improvement of the maximum degradation temperature of PLA. In general, CeF_3_ was beneficial to improving the thermal stability of PLA.

### 3.8. Dynamic Mechanical Analysis (DMA)

**Figure 11:**
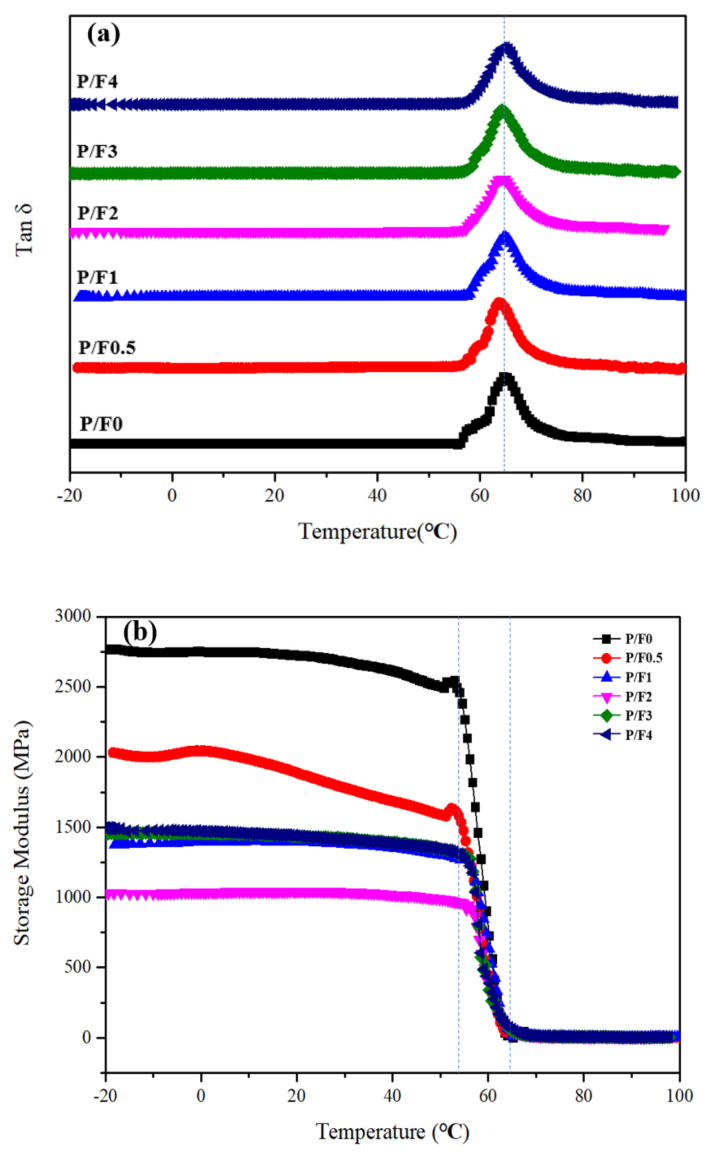
P/F film of (**a**) tan δ (**b**) storage modulus change diagram.

DMA was used to analyze the miscibility and modulus changes of the P/F films. Figure 11a,b show the dependence of loss factor (tan δ) on the temperature for PLA mixtures with different contents of CeF_3_. Only one tan δ peak was observed for P/F films, while pure PLA had a *T_g_* of 65 °C. Figure 11a,b show that the incorporation of CeF_3_ resulted in a small change in the glass transition temperature of PLA. The tan δ peaks of all the P/F films (near 65 °C) were between that of pure PLA. As shown in Figure 11b, the storage modulus (E′) of pure P/F films gradually decreased with the increasing CeF_3_ component, and the storage modulus (E′) of the P/F2 films was 1000 MPa, which was 63.6% lower than that of pure PLA. This suggested that P/F films showed a lower storage modulus than pure PLA from −20 to 60 °C, which showed that CeF_3_ had increased the flexibility of PLA, which was consistent with the test results of mechanical properties.

## 4. Conclusions

In this paper, P/F films were prepared, and the effects of different amounts of CeF_3_ on mechanical properties, visible light transmittance, thermal stability and crystallinity were studied. The results are as follows: The tensile strength and elongation at break of the P/F films increased first and then decreased with the increase in CeF_3_ content. The tensile strength of P/F1 was 59.92 MPa and the elongation at break of P/F2 was 2.53%, which was 8.76% and 78% higher than that of pure PLA, respectively. UV-vis transmittance analysis showed that CeF_3_ had little effect on the transparency of P/F films. When the content of CeF_3_ in PLA was 1 wt %, the visible light transmittance retention ratio of the P/F0 film was 82.36%. DSC analysis showed that CeF_3_ promoted the crystallization of PLA; compared to pure PLA, the crystallinity of P/F1 increased by 56%. TG analysis showed that the initial degradation temperature of the P/F2 films was 330.8 °C, and the maximum degradation temperature was 364.2 °C, which was 15.1 and 10.1 °C higher than that of pure PLA, respectively. DMA analysis showed that the addition of CeF_3_ could reduce the storage modulus of P/F effectively; the storage modulus (E′) of the P/F2 films was 1000 MPa, which was 63.6% lower than that of pure PLA, indicating that CeF_3_ had a toughening effect on the PLA film. As a result, CeF_3_ can improve the performance of PLA without affecting transparency, which had a potential application value in the field of food packaging.

## Figures and Tables

**Table 1:** Formulation of P/F films by mass fraction percentage.

Samples	PLA	CeF_3_
P/F0	100	0
P/F0.5	99.5	0.5
P/F1	99	1
P/F2	98	2
P/F3	97	3
P/F4	96	4

**Table 2:** Transmittance of P/F films in visible light with different amount of CeF_3_.

Sample	Visible Light Transmittance/%
P/F0	78.97
P/F0.5	72.19
P/F1	65.04
P/F2	52.58
P/F3	50.71
P/F4	42.62

**Table 3:** The phase transition parameters of P/F films.

Sample	$T_{cc}$ (°C)	$∆H_{c}$ (J/g)	$T_{m}$ (°C)	$∆H_{c}$ (J/g)	$X_{c}$ (%)
P/F0	116.25	10.97	172.92	28.70	19.0
P/F1	103.99	1.87	173.21	29.62	29.8
P/F2	109.44	4.25	172.16	26.92	27.2
P/F4	109.21	1.41	171.79	26.19	26.6

**Table 4:** TGA data of P/F films.

Samples	$T_{5\%}$ Weight Loss (°C)	$T_{max}$ Weight Loss (°C)	Reside at 500 °C (%)
P/F0	315.7	354.1	0.54
P/F1	318.5	357.6	1.53
P/F2	330.8	364.2	2.17
P/F4	308.4	355.7	4.52
